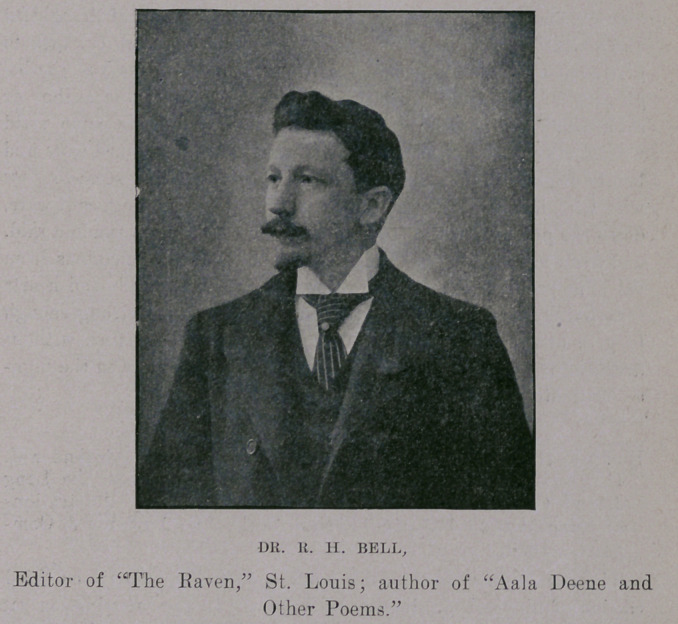# Books and Magazines

**Published:** 1900-03

**Authors:** 


We are indebted to Dr. Bell for a copy of his book. Not being a poet, we confess ourselves incompetent to give it a "review"—a criticism; but it strikes us as the work of a genius. It is as bold in expression as it is brilliant in conception. It has the ring of steel on steel, and fairly makes one's head swim—an old man's, we mean, by, I suppose, its power of bringing back the blood of youth (to the head, I mean). The book is a poem of passions,—"Love, love, love; it is a dizziness that will not let a puir mon gang about his business," or words to that effect. (See Burns.) Much of it reminds me of Swinburn, and it lays Ella Wheeler Wilcox in the shade, being free from the "sickly sentimental." Mr. Wallace Putnam Reed, the book critic of the *Atlanta Constitution*, says of the book and its talented author:

"Dr. Ralcy H. Bell is widely known among magazine and newspaper readers as a brilliant, rhythmical writer of prose and ringing verse. Moulton & Co., of Buffalo, N. Y., have recently published a pretty, dainty volume from his pen, entitled 'Aala Deene,' and Other Poems,' which cannot fail to attract the attention of the reviewers, and win a favorable verdict from a large circle of thoughtful and cultured readers. * * * It would be a delightful task to give even a fragmentary review of the volume mentioned at the beginning of this article, but it would not be just to the author or satisfactory to the reader to point out here and there some vivid word picture, daring conception, soul-stirring, impassioned lines and boldly original fancies, so powerfully phrased that they seem to glow with life, light and color as they run, shining and singing, as it were, down the printed page. It is not a book for a dashing, random raid, and doubtless no two reviewers would make the same selections from it. The poems in it appeal to those who have both heads and hearts —to living, flesh and blood men and women who are strong enough to give human nature a fair field and a fighting chance without dreading the consequences, when they feel that they are in the highest ultimate sense true to themselves."

The Bookman.—Dodd, Meade & Co., New York. Serial Story for 1900.

The editors of The Bookman consider themselves fortunate in having secured as their serial story for 1900 a novel by an American author, John Uri Lloyd, who, although unknown as yet as a writer of fiction, is believed to deserve a foremost place among the newer American novelists.

The Story is entitled "Stringtown on the Pipe," and it will be published in about ten numbers of The Bookman, beginning in March.

"Stringtown on the Pike" is a novel that none but an American could write. It is drenched with the American spirit and rooted in American traditions. It is a work that could only be produced by one who has brooded long and patiently over the types and forms which are unified into a drama of American life on a large scale. "Stringtown on the Pike" has its rise and progress and close in one little obscure and undiscovered corner of the land, a Kentucky village. It does all that Mary Wilkins and others have done for a narrow circle of American life, but it has a significance and sweep and human intensity which takes in the universe by touching life at the base.

The characters are well defined and distinctly wrought out. That of the Red-Haired Boy has a characteristic note and sturdy individuality that make him unusually attractive and strong. The heroine has that sort of elusive, shy, untamed nature whose next act can not be calculated upon, that puts her among that portion of her sex which is hard to be classified. The old villagers, the Judge, the Professor, the Clergyman, the Colonel, etc., impress one so vividly and clearly that one feels that they are drawn to the life. But of all the characters in the story none can be said to be so distinctly a creation of which any author might be proud as old Cupe. He is the great triumph of "Stringtown on the Pike." If for no other reason, this character would lift the book far above mediocrity and give it distinction and literary achievement worthy of a noble pen. Cupe, proud, kindly, dignified, last scion of an ancient African monarchy, is every inch the King he claims to be by heredity right. He dominates the story as does his fateful spell. He threads its situations and crowns its action in the climax of the novel.

In telling of the story Mr. Lloyd is simple, yet strong; lucid, yet forceful in diction; eschewing literary forms, yet falling naturally into a spontaneous narrative style that has a grace of its own.

"Stringtown on the Pike" is a story that will increase our pride and strengthen our faith in the existence of an American literature.

The Medical Complications, Accidents and Sequelae of Typhoid or Enteric Fever.—By Hobart Amory Hare, M. D., B. Sc., Professor of Therapeutics in the Jefferson Medical College of Philadelphia, Physician to the Jefferson Medical College Hospital, Laureate of the Medical Society of London, of the Academie Royale de Medicine de Belgique, etc. With a special chapter on the Mental Disturbances Following Typhoid Fever. By F. X. Dercum, M. D., Clinical Professor of Diseases of the Nervous System in the Jefferson Medical College. Octavo, 267 pages, 21 engravings and two full-page plates. Cloth, $2.40, net. Lea Brothers & Co., publishers, Philadelphia and New York. 1899.

This book deals with one of the most interesting subjects connected with the general practice of medicine. The unusual things with which the practitioner meets in his management of typhoid cases are not sufficiently brought out in the average text-book, hence this work is timely, and will appeal to every physician who feels a deep interest in his profession. Following an introduction of general considerations, the author discusses the varieties of onset, the aberrant symptoms, states or complications of the well developed stage of the disease; the complication of the period of convalescence, the conditions which ape typhoid and the duration and immunity to second attacks. The chapter devoted to the mental complications of typhoid closes the book, and it is one of the most instructive of all.

## Figures and Tables

**Figure f1:**